# Intravitreal Devices for the Treatment of Vitreous Inflammation

**DOI:** 10.1155/2012/126463

**Published:** 2012-09-05

**Authors:** John B. Christoforidis, Susie Chang, Angela Jiang, Jillian Wang, Colleen M. Cebulla

**Affiliations:** ^1^Retina Division, Department of Ophthalmology, The Ohio State University Wexner Medical Center, Columbus, OH 43212, USA; ^2^Department of Ophthalmology, The Ohio State University Wexner Medical Center, Columbus, OH 43212, USA

## Abstract

The eye is a well-suited organ for local delivery of therapeutics to treat vitreous inflammation as well as other pathologic conditions that induce visual loss. Several conditions are particularly challenging to treat and often require chronic courses of therapy. The use of implantable intravitreal devices for drug delivery is an emerging field in the treatment of vitreous inflammation as well as other ophthalmologic diseases. There are unique challenges in the design of these devices which include implants, polymers, and micro- and nanoparticles. This paper reviews current and investigational drug delivery systems for treating vitreous inflammation as well as other pathologic conditions that induce visual loss. The use of nonbiodegradable devices such as polyvinyl alcohol-ethylene vinyl acetate polymers and polysulfone capillary fibers, and biodegradable devices such as polylactic acid, polyglycolic acid, and polylactic-co-glycolic acid, polycaprolactones, and polyanhydrides are reviewed. Clinically used implantable devices for therapeutic agents including ganciclovir, fluocinolone acetonide, triamcinolone acetonide, and dexamethasone are described. Finally, recently developed investigational particulate drug delivery systems in the form of liposomes, microspheres, and nanoparticles are examined.

## 1. Introduction

The eye is a model organ for the implantation of devices that provide long-lasting infusion of a therapeutic agent. It is easily accessible for implantation of such a device and success of therapy is measurable objectively by direct visualization of the intraocular structures and by patient responsiveness. The treatment of posterior uveitis and vitreous inflammation usually involves a chronic course of therapy often over a period of years. Topical agents require frequent administration which is often impractical for patients. Sub-Tenon's and intravitreal steroid injections also can require frequent retreatment to adequately control disease. Treatment with powerful systemic corticosteroid and immunomodulatory agents most often have poor vitreous penetration and can be associated with severe systemic side effects [1]. Implantable devices offer an alternative therapeutic approach that can circumvent many challenges of these other modes of therapy.

The first implantable device for clinical use was developed in 1992 [2]. Vitrasert, a nonbiodegradable implant, delivers ganciclovir into the eye for the treatment of acquired-immunodeficiency-syndrome (AIDS)-related cytomegalovirus (CMV). Newer biodegradable implantable devices can provide sustained release of pharmacologics. More recently, there are serious investigations of biodegradable polymers that encapsulate particulate systems for long-lasting delivery of therapeutic nanoparticles which can be injected intravitreally.

In this review, current and investigational drug delivery systems for treating vitreous inflammation are described. These are summarized in Tables 1 and 2.

## 2. Drug Delivery Implant Polymers

### 2.1. Nonbiodegradable Devices

Nonbiodegradable devices require surgical implantation and contain a drug reservoir within a permeable polymer membrane. Although useful in some clinical settings, nonbiodegradable implants are not without limitations. Due to their large size a relatively large incision is required for implantation. Furthermore, these devices typically require removal and reimplantation of a second device once the drug has been completely released. The rate of drug release can be slowed by decreasing the surface area or increasing the thickness of the permeable membrane [3]. Complications associated with these devices include retinal detachment, vitreous hemorrhage, epiretinal membrane formation, and dissolution of the implant [4]. 

#### 2.1.1. Polyvinyl Alcohol-Ethylene Vinyl Acetate Polymers

These devices are made of polyvinyl alcohol (PVA), a permeable polymer functioning as a structural component, and ethylene vinyl acetate (EVA), a nonpermeable hydrophobic polymer used to restrict drug release. These devices are essentially inert, almost devoid of intraocular inflammatory response when implanted, but must be removed to prevent fibrous encapsulation after drug delivery is complete. The initial device of this type was originally formulated to contain 5-fluorouracil and placed subconjuctivally to prevent scarring following glaucoma filtration [5]. Subsequent devices have been used for intravitreal dexamethasone and cyclosporine implantation [6, 7]. The major clinical application of this device type was the ganciclovir (Vitrasert, Bausch and Lomb) implant, which was used frequently prior to the development of highly active antiretroviral therapy for HIV.

#### 2.1.2. Polysulfone Capillary Fiber

These devices are water impermeable and contain deep macrovoids which increase the surface area for drug release. It is permeable to both lipophilic and hydrophilic compounds and is well tolerated [7]. Polysulfone capillary fiber implants have only been used experimentally for carboxyfluorescein dye release and daunomycin in rabbit eyes [8, 9].

### 2.2. Biodegradable Devices

Biodegradable devices are particularly useful as intraocular implants since they do not need to be removed and have increased flexibility in their shape. They can be formulated as rods, discs, and microparticles [3].

#### 2.2.1. Polylactic Acid, Polyglycolic Acid, and Polylactic-Co-Glycolic Acid

Polylactic acid (PLA), polyglycolic acid (PGA), and polylactic-co-glycolic acid (PLGA) are the most studied synthetic biodegradable polymers. They are biocompatible, biodegradable and are FDA approved for drug delivery [10]. These polymers are widely used as suture materials, bone screws and pins, vascular grafts and stents, and surgical scaffolds for tissue regeneration. 

PGA is a semicrystalline polymer that is synthesized using toxic solvents limiting its potential for clinical use since any residual solvent may react with the drug or tissue [11]. PLA is a hydrophobic polymer that degrades more slowly than PGA. PLGA is a copolymer of PLA and PGA and is the most widely used biodegradable polymer for drug delivery. The ratio of PLA to PGA can be adjusted to modulate the rate of polymer degradation. The rate of drug release depends on the total surface area of the device, the percentage of loaded drug, the water solubility of the drug, and the speed of polymer degradation for human immunodeficiency virus [12]. 

There are three phases of drug release in these types of polymers.

1. Initial burst from the surface of the implant.
2. Diffusion phase during biodegradation of the polymer.
3. Final burst from the disintegration of the implant.

The initial burst is followed by a longer steady drug release and is well suited for diseases that require an initial loading dose followed by tapering. However, the first and last phases release higher drug concentrations and potential toxic effects at these levels need to be considered. Blending polymers with different molecular weights can reduce the final drug burst and result in a more stable agent release [13, 14]. Examples of PLGA devices include dexamethasone (Ozurdex) and indomethacin.

#### 2.2.2. Polycaprolactones

Polycaprolactone (PCL) is a polymer of *ε*-caprolactone, a semi-crystalline and hydrophobic compound that is biodegradable and biocompatible. It is widely used in the biomedical industry (e.g., Monocryl suture, artificial skin, and osteosynthetic material). It is very slowly degraded in the human body by hydrolysis of its ester linkages and its fragments are phagocytized [15]. When the implants are immersed in water, there is dissolution leaving pores in the PCL, allowing for a long-term, well-controlled steady release rate over a period of greater than one year [16, 17]. Intravitreal PCL implants with 5-fluorouracil has been investigated for the prevention of proliferative vitreoretinopathy [18]. Intravitreally-placed PCL devices containing dexamethasone delivers the drug in a controlled and prolonged manner for at least 55 weeks. At 55 weeks, 79% of drug was still present in the implant. It was found to be very well tolerated in rabbit eyes with no sign of anterior or posterior segment inflammation [19]. PCL devices containing triamcinolone acetate have also been implanted in the subretinal space of rabbit eyes and was found to be well- tolerated by retinal tissue, releasing the drug for at least 4 weeks without an inflammatory response [20]. PCL can also be mixed with other polymers, usually more hydrophilic than PCL, to form copolymers which degrade at faster rates. These have been used experimentally for drug delivery of cyclosporine and tacrolimus [21, 22].

#### 2.2.3. Polyanhydrides

Polyanhydrides are a class of biodegradable polymers that degrade by surface erosion into biocompatible monomers that are then metabolized and removed from the body [23]. Surface erosion provides a more controlled drug release compared to drugs that are released by bulk erosion, making them useful as drug delivery devices. There are several classes of polyanhydrides including aliphatic, unsaturated, and aromatic. Aliphatic polyanhydrides degrade in a few days while some aromatic polyanhydrides degrade over few years. Degradation rates of copolymers of aliphatic and aromatic polyanhydrides vary between these extremes and this feature of polyanhydrides gives an opportunity for making a drug delivery system which can provide the release of drugs for a desired time length of treatment [24].

The most frequently used is a copolymer of the 1,3-bis(carboxyphenoxypropane) (PCPP) and sebacic acid (SA). PCPP is aromatic and hydrophobic and by itself has a long lifetime of over 3 years, while SA is aliphatic and hydrophilic with a lifetime of a few days. Copolymerization with SA reduces the lifetime to a few days [25]. The 80 : 20 copolymer has been FDA approved for intracranial delivery of carmustine (Gliadel) for treatment of brain tumors, and intravenous delivery for treatment of recurrent Hodgkin's lymphoma and multiple myeloma. In ocular use they have been investigated in the delivery of 5-fluorouracil, taxol, and etoposide for experimental glaucoma filtration surgery in a non-human primate model [26, 27].

### 2.3. Clinically Used Intravitreal Implants

#### 2.3.1. Ganciclovir

**Figure 1:**
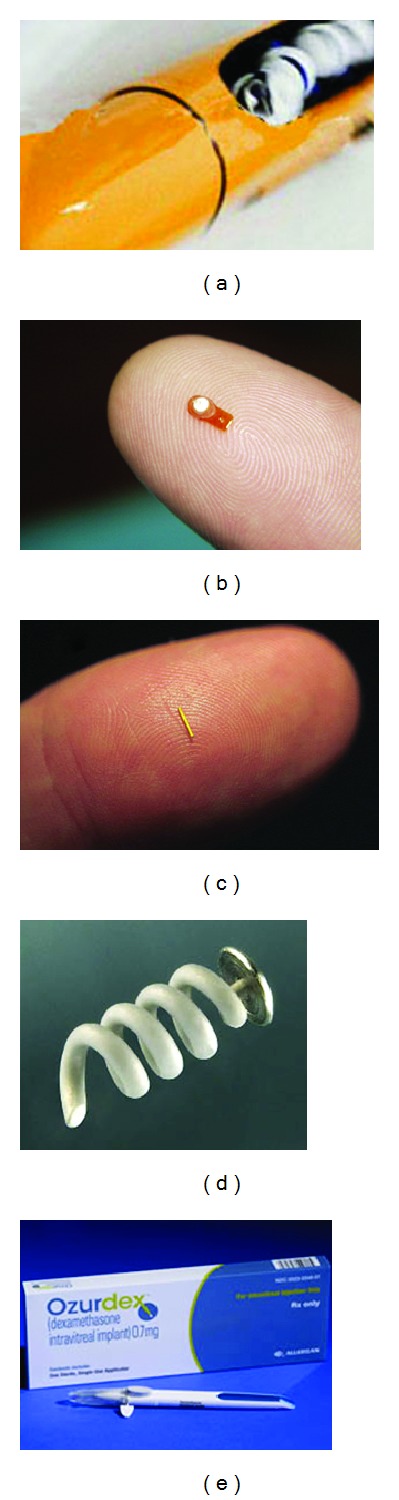
Intravitreal devices. (a) Vitrasert, image courtesy of Bausch & Lomb. (b) Retisert, image courtesy of pSIVIDA. (c) Medidur, image courtesy of pSIVIDA. (d) I-vation, image courtesy of SurModics, Inc. (e) Ozurdex, image courtesy of Allergan, Inc.

Vitrasert (Bausch & Lomb, Rochester, NY) is a PVA-EVA reservoir implant consisting of a pellet containing at least 4.5 mg of ganciclovir as the active ingredient and 0.25% magnesium stearate as the inactive ingredient with a ganciclovir release rate of 1 mcg/hour (Figure 1(a)). The EVA limits the surface area of ganciclovir. A 5-6 mm scleral incision is made at pars plana and after trimming away any prolapsed vitreous, the device is implanted into the vitreous cavity. It is sutured in place on the sclera prior to closing the sclera and overlying conjunctiva. It is removed if another ganciclovir implant is placed (usually after 6 months) or if there are any complications such as endophthalmitis or retinal detachment. Vitrasert offers superior control of retinitis over systemic ganciclovir therapy [28]. The Vitrasert disc is composed of outer and inner permeable PVA layers surrounding a discontinuous hydrophobic EVA film. The device allows diffusion of fluid into the device dissolving the drug pellet, which then diffuses into the vitreous at a constant rate [2].

#### 2.3.2. Fluocinolone Acetonide

Retisert (Bausch & Lomb, Rochester, NY) is a tablet containing 0.59 mg of fluocinolone acetonide that is coated with nonbiodegradable PVA and silicon laminate (Figure 1(b)). It is 5 mm long, 2 mm wide and 1.5 mm thick with a release rate of 0.3–0.6 mcg/d over a period of 30 months. It is inserted into the vitreous cavity and sutured to the sclera through a pars plana surgical technique similar to Vitrasert. In April 2005 it became the first FDA-approved device for use in the treatment of chronic noninfectious posterior uveitis [29].

In clinical studies Retisert was found to significantly reduce inflammation and lower intravitreal vascular endothelial growth factor (VEGF) levels. In patients with noninfectious posterior uveitis treated with Retisert, the recurrence rate of uveitis was reduced from 62% before treatment to 4%, 10%, and 20% at 1, 2, and 3 years, respectively following treatment [30, 31]. Despite the excellent reduction of uveitis, the complication rate was high. At 34 weeks, 51% of patients had an increased intraocular pressure (IOP) that required pressure lowering agents. At 3 years, 78% required pressure lowering agents and approximately 40% required glaucoma filtering surgery. In addition, 100% of phakic patients developed cataract formation within 3 years of implantation. Other side effects included hypotony (6.1%), retinal detachment (2.9%), endophthalmitis (0.4%), and the need for explantation at 2 years (3.6%) [31, 32].

Recently, the Multicenter Uveitis Steroid Treatment (MUST) trial compared the relative effectiveness of systemic therapy and fluocinolone acetonide implant for the treatment of noninfectious uveitis in 479 eyes over 2 years. It found that both treatment groups were effective and neither group was superior to the other in improving visual acuity. Systemic therapy was well tolerated while the implant group had an 80% risk of cataract surgery and 61% required treatment for elevated intraocular pressures [33].

#### 2.3.3. Iluvien (Fluocinolone Acetonide)

Illuvien (Alimera Sciences Inc., Alpharetta, GA; pSivida Inc., Watertown, MA) is an injectable nonbiodegradable intravitreal implant containing fluocinolone acetonide (Figure 1(c)). It is 3.5 mm long and 0.37 mm wide and releases fluocinolone acetonide at a rate of 0.2 mcg or 0.5 mcg per day over 18–36 months. It is inserted with a 25 gauge needle. Phase III clinical trials for diabetic macular edema (DME) were recently concluded.

The fluocinolone acetonide for diabetic macular edema (FAME) study group tested the low dose 0.2 mcg insert and the high dose 0.5 mcg insert against a sham implant. At 24 months, 28% of those receiving either dose had an improvement of ≥15 in best-corrected visual acuity (BCVA) letters compared to 16% of those in the control. At 36 months, it was 33.0% in the low dose and 31.9% in the high dose compared with 21.4% in the sham. Increased incidence of cataracts was seen in implanted eyes but long-term vision was not compromised. Increase in intraocular pressure was also a concern with implantation of the device. At 36 months, 4.8% of those receiving the low dose implant required glaucoma surgery but visual outcome was not impacted when compared to those who did not require incisional surgery. These results show promise in DME patients who otherwise have limited effective treatment options [34]. In addition to its use in DME patients, phase II trials for the treatment of exudative age-related macular degeneration (AMD) and retinal vein occlusion are also being conducted.

#### 2.3.4. Triamcinolone Acetonide

**(1) I-vation**

(SurModics, Eden Prairie, MN) is a 0.4 mm × 0.21 mm titanium helical nonbiodegradable implant that contains 0.925 mcg triamcinolone acetonide (Figure 1(d)). Triamcinolone is coated with polybutyl methacrylate and polyEVA. It is intended for a sustained delivery of 2 years. The helical design increases the surface area for drug release and stabilizes the device onto the sclera [35]. It was recently found to be effective in the treatment of diabetic macular edema after 24 months in a Phase I clinical trial although all phakic patients developed visually significant cataracts and increases in intraocular pressure occurred in 50% of eyes [36].

**(2) Verisome**

Verisome (Ramscor, Inc., Menlo Park, CA) is a nonpolymer-based intraocular drug delivery system that provides long-acting intravitreal drug therapy. It can be injected through the pars plana using a 30 gauge injector. Triamcinolone has been used investigationally providing a mean vitreous level of 1.1 mcg/mL for up to 1 year [37].

#### 2.3.5. Dexamethasone

**(1) Ozurdex**

Ozurdex (formerly Posurdex Allergan Inc., Irvine CA) is a rod-shaped 6.5 × 0.45 mm pellet composed of a mixture of dexamethasone as the active pharmaceutical ingredient (API) and biodegradable PLGA (Figure 1(e)). Although dexamethasone has a short half-life relative to triamcinolone, it is 20 and 5 times more potent than fluocinolone and triamcinolone, respectively (Table 3) [38].

Ozurdex is placed intravitreally through the pars plana with an injector using a 22-gauge needle device. The insert contains 0.7 mg dexamethasone and provides peak doses for 2 months initially followed by lower doses for up to 6 months. Ozurdex received FDA approval in June 2009 for the treatment of macular edema associated with retinal vein occlusion, and in September 2010, it became the second FDA-approved therapeutic agent for the treatment of noninfectious posterior uveitis.

In a 26-week, multicenter, double-masked, randomized clinical study in which 229 patients were randomized in a 1 : 1 : 1 ratio receiving 0.70 mg Ozurdex (*n* = 77), 0.35 mg Ozurdex (*n* = 76), or sham injection (*n* = 76). Eighty-one percent of patients had intermediate uveitis. At the eighth week primary endpoint, 47%, 36% and 12% of patients had no vitreous inflammation. The response was maintained at week 26. In addition, both treatment groups achieved a 3-line improvement in visual acuity and reduced central macular thicknesses on ocular coherence tomography at 8 weeks that was statistically significant compared to the sham group. The complication rates were not found to be significant. Twenty-three percent of eyes in the 0.7 mg Ozurdex group required IOP-lowering agents and none needed surgical intervention for glaucoma. Cataract formation was seen in 15% in the 0.7 mg group, 12% in the 0.34 mg group, and 7% in the sham group [39, 40].

**(2) Surodex**

Surodex (Oculex Pharmaceuticals, Sunnyvale, CA) is a 1.0 × 0.4 mm PLGA pellet that provides sustained release of dexamethasone after insertion into the anterior chamber. It is primarily targeted to reduce post-cataract surgery inflammation for 7–10 days [41].

### 2.4. Experimental Intravitreal Implants

#### 2.4.1. Cyclosporine

Cyclosporine A placed in the deep sclera adjacent to the suprachoroidal space has been found to be effective in controlling uveitis in an equine recurrent uveitis model [42]. In a chronic uveitis rabbit model, 2 mg cyclosporine A conjugated to a PCL/PLGA copolymer was found to be significantly more effective than oral cyclosporine [20].

#### 2.4.2. Indomethacin

PLGA discs containing 7 mg of indomethacin released over 3 weeks was evaluated in a rabbit model. Although postoperative inflammation was decreased there was no significant decrease in posterior capsular opacification of the lens [43]. 

#### 2.4.3. Particulate Drug Delivery Systems

The long-term drug delivery of small scale biodegradable devices has been recently investigated in experimental studies. These include liposomes, microspheres, and nanoparticles.

**(1) Liposomes**

**Figure 2:**
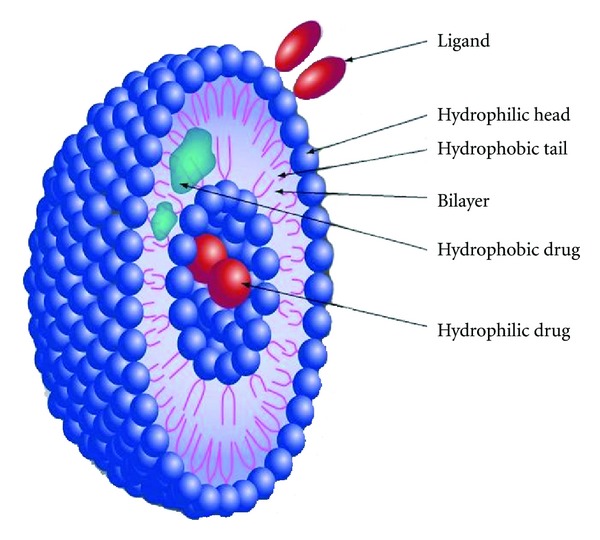
Liposome and its different drug-loading and surface functionalization modalities. (Courtesy of Nanomedicine (2010) Future Medicine Ltd).

Liposomes are spherical liposomal structures, about 0.01 to 10 *μ*m in diameter (Figure 2). They are formed of a vesicular lipid bilayer separated by water or an aqueous buffer compartment [44]. They can circumvent cell membrane barriers and protect drugs from metabolic or immune attack. Since the phospholipid bilayers are naturally occurring, they are biocompatible and minimize toxicity and immunogenicity.

Liposomes are colloidal particles made of phospholipids that encapsulate hydrophobic or hydrophilic therapeutic agents. They often contain inner aqueous spaces where hydrophilic enzymes remain soluble and hydrophobic outer layers that allow passage through natural membrane barriers.

Currently verteporfin (Visudyne, QLT Inc. Vancouver BC, Canada) is the only liposomal drug that is FDA approved for use in the eye for the treatment of predominantly classic wet AMD. Liposomal amphoterecin B (AmBisome, Gilead Sciences, Foster City, CA) is used off-label for the treatment of fungal endophthalmitis and has been found to exhibit fewer side effects than the nonliposomal forms allowing for higher dosages (up to 30 micrograms) to be injected intravitreally [45].

**(2) Particulate Ocular Drug Delivery Systems**

**Figure 3:**
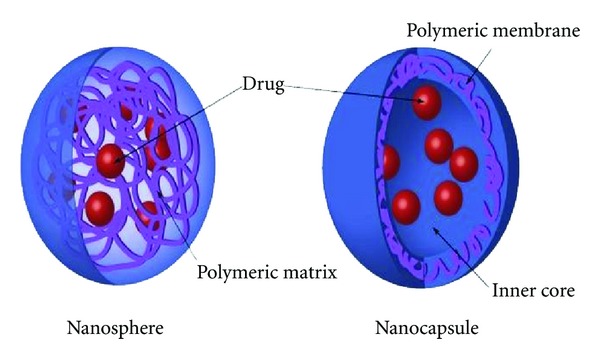
The two main types of polymeric nanoparticles known as nanosphere (matrix system) and nanocapsule (reservoir system) with different drug-loading modalities. (Courtesy of Nanomedicine (2010) Future Medicine Ltd).

Particulate Ocular Drug Delivery Systems include nanoparticles and microparticles. Although the distinction is often not consistent, nanoparticles are considered to be between 10 and 1,000 nm in size and microparticles 1 to 1,000 *μ*m in diameter [46]. Nanoparticles and microparticles are subdivided into nanospheres and microspheres which are a polymer-drug combinations where the drug is homogenously dispersed in the polymeric matrix, and nanocapsules/microcapsules, in which the drug particles or droplets are entrapped in a polymeric membrane (Figure 3).

**(3) Microparticles**

Microparticles are similar to liposomes in shape, size, and route of administration. However, nanoparticles offer several advantages over microsomes such as higher stability and larger drug-loading capabilities. Polymers such as PLGA and PLA are widely used for nanoparticle drug delivery systems. Surface polymer modifications also provide greater protection of the drug against degradation and phagocytosis by macrophages [47]. Although there are no currently used FDA approved microparticle devices, a wide variety of therapeutic agents are being investigated to improve the cellular penetration and allow long-term delivery using microsphere and nanosphere technology (Table 4).

**(4) Microspheres**

Microspheres have been developed for sustained ocular delivery of therapeutic agents such as progesterone, adriamycin, and pegaptanib [48–50]. Microspheres composed of chitosan, a natural biodegradable polymer, have been used for transcorneal acyclovir delivery [51]. A sustained release of microsphere-encapsulated cyclosporine was found to be present compared to cyclosporine solution [52].

**(5) Nanoparticles**

Nanoparticles have been used experimentally with several agents. Tamoxifen (PEG coated) was found to be effective in the treatment of experimental autoimmune uveitis in a rat model [53]. Intravitreally injected nanoparticles containing ganciclovir and acyclovir have been studied in a rabbit model with steady drug concentrations, but were found to be associated with cataract formation and flare [54]. Scleral injections of pigment epithelium-derived factor (PEDF) resulted in increased PEDF expression in the retina and retinal pigment epithelium and resulted in significant reductions of choroidal neovascularization in mouse and pig models [55, 56]. In a Phase I clinical trial, recombinant adeno-associated viral mediated expression of (rAAV-PEDF) was administered intravitreally in patients with exudative AMD. Although this resulted in transient intraocular inflammation (25%) and IOP elevations (21%), no other adverse events were seen and the majority of the patients achieved stable or improved visual acuity [57].

## 3. Summary

In conclusion, the eye is well suited for local delivery of therapeutics to treat vitreous inflammation as well as other pathologic conditions that induce visual loss. However, there are some unique challenges in designing local ocular drug delivery devices, which include implants, polymers, and micro- and nanoparticles. An integrated approach involving biomedical engineering, molecular biology, immunology, pathology, and pharmacology will continue to be critical to designing optimal devices for ocular inflammation and other diseases.

## Figures and Tables

**Table 1:** Drug delivery implant polymers.

Material	Properties	Clinical application
Nonbiodegradable devices		

Ethylene vinyl acetate	Nonpermeable, hydrophobic	Vitrasert implant, intravitreal dexamethasone, and cyclosporine
Polysulfone capillary fiber	Water impermeable; increases surface area for drug release	Used experimentally for carboxyfluorescein dye release and daunomycin in rabbit eyes
Polyvinyl alcohol	Permeable	Vitrasert implant, intravitreal dexamethasone, and cyclosporine

Biodegradable devices		

Polyanhydrides	Degrade by surface erosion into biocompatible monomers	5-fluorouracil, taxol, and etoposide
Polycaprolactone	Semicrystalline, hydrophobic	5-fluorouracil, dexamethasone, and triamcinolone implants
Polyglycolic acid	Semicrystalline; synthesized using toxic solvents	
Polylactic acid	Hydrophobic; degrades more slowly than polyglycolic acid	
Polylactic-co-glycolic acid	Copolymer (adjustable ratio) of polyglycolic and polylactic acid	Dexamethasone (Ozurdex), indomethacin

**Table 2:** Characteristics of intravitreal devices.

Device	Materials	Active agent	Duration of drug release	Diseases
Nonbiodegradable devices				

I-vation	Drug-polymer-coated nonferrous alloy helix (polybutyl methacrylate/polyvinyl alcohol; bravo drug delivery polymer matrix)	Triamcinolone acetonide (1–3 *μ*g/day)	2 years	Investigational: diabetic macular edema phase 2b trial suspended in 2008
Illuvien/medidur	Polyvinyl alcohol (with silicone bioadhesive in low-dose version)	Fluocinolone acetonide (0.59 mg; 0.2–0.5 *μ*g/day)	18–30 months	Investigational: diabetic macular edema (phase 3)
Retisert	Silicone/polyvinyl alcohol	Fluocinolone acetonide (0.59 mg)	Up to 3 years	FDA approved for the treatment of uveitis. Investigational: diabetic macular edema, retinal vein occlusion
Vitrasert	EVA/polyvinyl alcohol	Ganciclovir (4.5 mg)	5 to 8 months	Implantable reservoir system

Biodegradable devices				

Ozurdex	Polylactic-co-glycolic acid	Dexamethasone (0.7 mg)	6 months	DA approved for the treatment of macular edema following branch or central retinal vein occlusion. Investigational: diabetic macular edema, uveitis
Surodex	Polylactic-co-glycolic acid, hydroxypropyl methylcellulose	Dexamethasone (60 *μ*g)	7–10 days	Investigational in the USA: postoperative inflammation following cataract surgery (phase 3). Regulatory approvals in Singapore, China, Mexico

**Table 3:** Comparison of corticosteroid properties.

Steroid	Water solubility (*μ*/mL)	Half-life	Relative potency
Triamcinolone acetonide	21	18 days	1
Fluocinolone acetonide	50	1.3–1.7 hours	0.4 *x*
Dexamethasone	100	3–5 hours	3–5 *x*

**Table 4:** Comparison of intravitreal implants for the treatment of noninfectious uveitis [40].

	0.59 mg fluocinolone acetonide (FA) implant	0.7 mg dexamethasone (DEX) implant
Administration	Operating room	Officebased
Matrix	Non-biodegradable	Biodegradable
Duration of effect	30 months	6 months
Improvement of >15 letters (% eyes)	21% by week 34	38% by week 26
Rescue medications (% eyes)	25.4% by week 34	22% by week 26
Glaucoma surgery (% eyes)	30.6% by month 24	0.5% by month 6
Cataract surgery (% eyes)	89.4% by month 24	4% by month 6
